# Genomic variation in macrophage-cultured European porcine reproductive and respiratory syndrome virus Olot/91 revealed using ultra-deep next generation sequencing

**DOI:** 10.1186/1743-422X-11-42

**Published:** 2014-03-04

**Authors:** Zen H Lu, Alexander Brown, Alison D Wilson, Jay G Calvert, Monica Balasch, Pablo Fuentes-Utrilla, Julia Loecherbach, Frances Turner, Richard Talbot, Alan L Archibald, Tahar Ait-Ali

**Affiliations:** 1 The Roslin Institute and Royal (Dick) School of Veterinary Studies, University of Edinburgh, Easter Bush, Edinburgh EH25 9RG, UK; 2 Zoetis Inc.,Global Biologics Research, 333 Portage St, Kalamazoo, MI 49007, USA; 3 VMRD Olot Zoetis, Ctra. EU Regional Vaccines Group, Camprodon s/n Finca "La Riba", 17813 Vall de Bianya, Girona, Spain; 4 Edinburgh Genomics, University of Edinburgh, Easter Bush, Edinburgh EH25 9RG, UK

**Keywords:** PRRSV, Microevolution, Variant spectra, Ultra-deep next generation sequencing

## Abstract

**Background:**

Porcine Reproductive and Respiratory Syndrome (PRRS) is a disease of major economic impact worldwide. The etiologic agent of this disease is the PRRS virus (PRRSV). Increasing evidence suggest that microevolution within a coexisting quasispecies population can give rise to high sequence heterogeneity in PRRSV.

**Findings:**

We developed a pipeline based on the ultra-deep next generation sequencing approach to first construct the complete genome of a European PRRSV, strain Olot/9, cultured on macrophages and then capture the rare variants representative of the mixed quasispecies population. Olot/91 differs from the reference Lelystad strain by about 5% and a total of 88 variants, with frequencies as low as 1%, were detected in the mixed population. These variants included 16 non-synonymous variants concentrated in the genes encoding structural and nonstructural proteins; including Glycoprotein 2a and 5.

**Conclusion:**

Using an ultra-deep sequencing methodology, the complete genome of Olot/91 was constructed without any prior knowledge of the sequence. Rare variants that constitute minor fractions of the heterogeneous PRRSV population could successfully be detected to allow further exploration of microevolutionary events.

## Findings

Porcine Reproductive and Respiratory Syndrome virus (PRRSV) is the causative agent of a significant disease of the domestic pig (*Sus scrofa*) with global consequences. The severity of PRRSV infection ranges from subclinical to lethal and it affects pigs in both growing and reproductive stages. The virus has a positive-sense 15 kb RNA genome and its genetic diversity has been well characterised within and between European and North American strains [1]. Extensive viral genetic heterogeneity may have contributed towards the observed variations between PRRSV isolates and clones in term of virulence, interactions with the immune system, and antigenic properties of viral proteins. Such a broad diversity indeed poses serious challenges to diagnostics and control measures.

Most previous studies of PRRSV genetic diversity have been restricted to the ORF5 and ORF7 sequences of type 2 “North American-like” viruses that also include the Asian variants. Only 14 of the 303 completed PRRSV genomes in Genbank belong to genotype 1. Furthermore, studies have shown that PRRSV mutates rapidly and multiple intra-strain variants can coexist in individually infected pigs [2]. The extensive genetic diversity displayed by PRRSV and other RNA viruses such as HIV and influenza reflects the error prone nature of RNA polymerases, which lack a proofreading function [3,4].

To identify PRRSV quasispecies, previous studies have employed conventional methodologies including reverse-transcription, PCR, cloning and Sanger sequencing of a subset of PRRSV structural and non-structural proteins [2,5]. More recently next-generation sequencing (NGS) of fragments generated by long range RT-PCR has been used to characterise multiple PRRSV genomes [6]. However, this approach relies upon prior knowledge of the target sequence and the assumption that the PCR primer binding sites are non-variable. Here we describe an approach which requires no prior knowledge of the target sequences and which should enable the detection of low-frequency nucleotide variants and hence provides a snapshot of the microevolution in the entire viral population.

We analysed the intra-strain sequence diversity of low passage PRRSV Olot/91 strain, passaged exclusively on primary porcine alveolar macrophages (PAM). First reported in Spain in 1991, Olot/91 is the parent strain of the commercial Suvaxyn PRRSV inactivated vaccine which is used in Spain and Portugal. Only a partial sequence of 3,383 nt [GenBank:X92942], that covers ORFs 2-7 and the 3'-UTR of this strain has previously been published [7].

**Figure 1:**
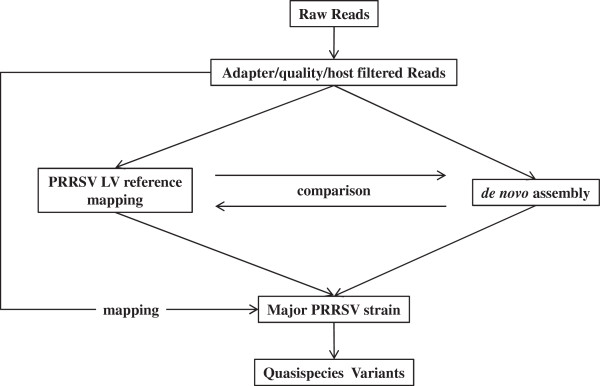
**Schema of the strategy used to analyse the next generation sequencing data.** Raw data were quality filtered before being either mapped to a reference genome or assembled *de novo*. The filtered reads were then mapped back onto the newly constructed genome to search for rare variants from the quasispecies population.

**Figure 2:**
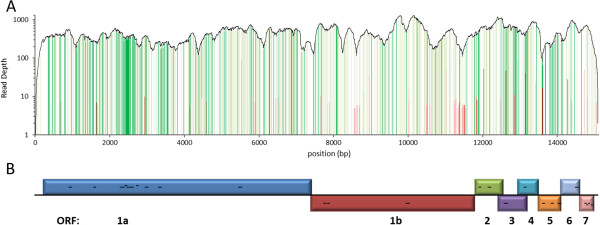
**PRRSV genomic variant spectra. (A)** Note that the read depth is plotted on a log scale. The black solid curve depicts the coverage of the mapped Olot/91. The green bars show the locations of the Olot/91's variants when compared to the reference LV genome while the red bars are mixed variants in the viral population. Darker bars are variants that led to non-synonymous changes. **(B)** Genomic organisation of PRRSV. B- and T-cell epitopes distributed across the ORFs were represented with black horizontal bars.

To reduce potential complications with sub-genomic RNA, the virus was grown in porcine alveolar macrophages for 5 days and viral particles were pelleted from cell culture supernatant through a 30% sucrose cushion. Viral RNA was then prepared using BioSprint 96 DNA Blood kit (Qiagen) and sequencing libraries produced using the Illumina TruSeq RNA sequencing library preparation kit, with a modification eliminating the initial mRNA isolation steps. A total of 7.56 million 225 bp reads were generated on a Illumina MiSeq machine. They were analysed following a 2-step strategy whereby the consensus sequence of the major Olot/91 strain was first obtained and then used to call rare variants present in the evolving PRRSV viral population (Figure 1). To account for potential contamination with host DNA, the raw reads were initially filtered against the pig genome (Sscrofa10.2 [8]) using the program Best Match Tagger (v1.1.0) [9] which removed about 88% of the reads. The remaining reads then underwent a stringent quality filtering whereby the first 5 bp of the 5' ends were trimmed to remove any remnant adapter sequences; reads with ambiguous bases and Phred scores lower than 20 were discarded using Sickle (v1.2) [10] and the 3' ends were further scanned for run-through adapter sequences using Scythe (v0.991b) [11]. The final high quality reads (4.3%) were next either mapped against the reference Lelystad (LV) genome [Genbank:M96262] [12] using BWA (v0.6.2) [13]/Samtools (v0.1.18) [14] or assembled *de novo* with Velvet (v1.2.9) [15]. Variants used to call the 15,111 nt consensus sequence in the mapped major Olot/91 strain were determined as those having allele frequencies higher than 0.7 by the program Lofreq (v0.6.0) [16]. A total of 742 single nucleotide variants were identified that differed from the reference LV strain (Figure 2, Additional file 1: Table S1). Of these, 180 resulted in non-synonymous changes in coding sequences. No insertions or deletions were found. For the *de novo* assembly, contigs from two kmer values of 161 and 195 were merged with Lasergene's SeqMan Pro (v10.1) to yield a single contig of 15,082 nt. Both the mapped consensus sequence and the *de novo* assembled contig were aligned against each other to ensure the accuracy of the genome. These sequences were exactly identical to each other and the full length Olot/91 genome has now been deposited in Genbank with the accession number KF203132. This full length Olot/91 contains 8 SNPs and a 3 nt insertion when compared with the published partial Olot/91 sequence. However, this PAM-passaged virus only shares 96.8% similarity with the MARC-145 cell-adapted PRRSV Olot/91 strain (Genbank:KC862570) [6]. The differences may represent the molecular basis for the adaption to growth on MARC-145 cells which are of African Green Monkey origin [17].

**Figure 3:**
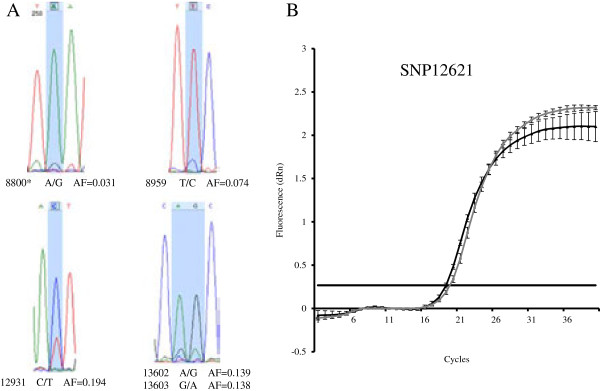
**Variant validation using Sanger sequencing and realtime PCR. (A)** Minor peaks representative of the quasispecies' genotypes could be seen under the highlighted major peaks from Olot/91. *The 3% low frequency variant can be further confirmed by the raw chromatogram (Additional file 2: Figure S2). **(B)** Variants from the main Olot/91 and its associated quasispecies resulted in different Ct curves.

The same set of cleaned reads was then remapped as previously onto the newly generated Olot/91 genome to an average depth of approximately 530×. Eighty-eight low-frequency single-nucleotide variants representing the genotypic makeup of the heterogeneous PRRSV population were identified, of which 16 represented non-synonymous changes in coding sequences (Figure 2, Additional file 1: Table S1). Among the 22 low frequency variants that overlap with the major Olot/91 versus reference LV strain variants, 17 have evolved into genotypes identical to the reference strain. The frequency of intra-strain variants detectable in this study ranged from 1 to 20% with each supported by a minimum of 3 reads. A subset of these low-frequency variants was validated using two alternative methods; Sanger sequencing and real-time PCR. Reverse transcription was performed as described previously [18]. Briefly, one microgram of the Olot/91 RNA was reverse transcribed using a TaqMan kit (Applied Biosystems, Foster City, CA). For Sanger sequencing, PCR products encompassing the variants were sequenced in both directions using BigDye terminator v3.1 Cycle Sequencing kit (Applied Biosystems). Variants with frequencies as low as 3% could be successfully identified by the overlapping peaks (Figure 3A and Additional file 2: Figure S1). Major and minor allele specific real-time PCRs employing Platinum SYBR Green PCR SuperMix UDG (Invitrogen, Paisley, UK) were run on a Stratagene MX3000P (Stratagene). Samples were run in triplicate, beta-actin was utilized as the housekeeping gene and results were calculated as described previously [18]. Again, the main Olot/91 sequence and the variants present in the related mixed population could both be successfully detected (Figure 3B).

While variants in the major Olot/91 strain do congregate on known highly hypervariable B- and T-cell epitopes of type I PRRSV (Figure 2 and Additional file 3: Figure S2) [19-23], most of the variants representative of the probable quasispecies population map to the *nsp12 and gp2a* genes of ORF1b and ORF2 respectively (Figure 2); suggesting a higher microevolutionary rate at this region under the investigated culture conditions. In addition, the predicted short signal peptide of GP2a was found to harbour a high concentration of non-synonymous variants and a potential N-glycosylation site was created from a residue change (S37N) on GP5. Further analysis, like molecular modelling, may be necessary to decipher the potential impacts of these evolving variants have on such functions as interactions between these proteins and other viral or host cellular proteins.

Using ultra-deep NGS we have constructed the complete sequence of the PRRSV Olot/91 genome cultured in their natural host - porcine alveolar macrophages - and identified single-nucleotide variants present in the associated viral population. Further investigation using this methodology will help to establish if a link exists between the microevolutionary dynamics and pathogenesis of PRRSV viral strains. However, it is also important to note that the exact nature of the pathogenesis cannot be truly identified until the viral haplotypes within the quasispecies population can be reconstructed with confidence [24].

## Competing interests

The authors declare that they have no competing interests.

## Authors’ contributions

ZHL carried out all the bioinformatics analyses and drafted the manuscript. AB participated in the discussion of data analysis. JGC and MB provided the samples. ADW cultured the virus, purified the viral RNA and performed the real time PCR. PFU constructed the RNA libraries and ran the next-generation sequencing. JL and FT prepared the raw Illumina data. RT designed the sequencing run and collaborated in drafting the manuscript. ALA conceived the study and collaborated in drafting the manuscript. TAA conceived/ designed/coordinated the study and drafted the manuscript. All authors read and approved the final manuscript.

## Supplementary Material

Additional file 1: Table S1 Annotation of Single-nucleotide variants detected in the mixed Olot/91 population. (A) SNPs detected in comparison to the reference LV strain. (B) Rare variants detected in comparison to the major Olot/91 strain. Click here for file

Additional file 2: Figure S1 Unprocessed raw chromatogram depicting the unique low-frequency variant in the mixed Olot/91 population. The unique black G peak (circled) stands up among the noise shoulders of A (green) and T (red). Click here for file

Additional file 3: Figure S2 Sequence Entropy of type I European PRRSV Strains. Multiple sequence alignment of the genomes [Olot/91-GenBank:KF203132, GenBank:M96262, GenBank:GU737264, GenBank:A26843, GenBank:GQ461593, GenBank:FJ349261, GenBank:DQ489311, GenBank:JF802085, GenBank:GU047344, GenBank:GU047345, GenBank:AY588319, GenBank:AY366525, GenBank:GU067771, GenBank:EU076704, GenBank:DQ864705] were generated with MUSCLE and entropy analysed with Hyphy [25]. Higher entropies denote regions with higher variability. Click here for file
