# Supplementary figures and images for: Genomic variation in macrophage-cultured European porcine reproductive and respiratory syndrome virus Olot/91 revealed using ultra-deep next generation sequencing
Source: Virol J. 2014 Mar 4;11:42. doi: 10.1186/1743-422X-11-42 (PMC3945042; doi:10.1186/1743-422X-11-42)

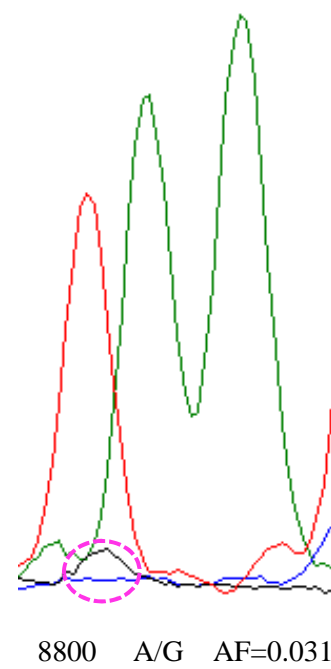

**Figure S1**

Supplement: Additional file 2: Figure S1 — Unprocessed raw chromatogram depicting the unique low-frequency variant in the mixed Olot/91 population. The unique black G peak (circled) stands up among the noise shoulders of A (green) and T (red). [file 1743-422X-11-42-S2.pdf]

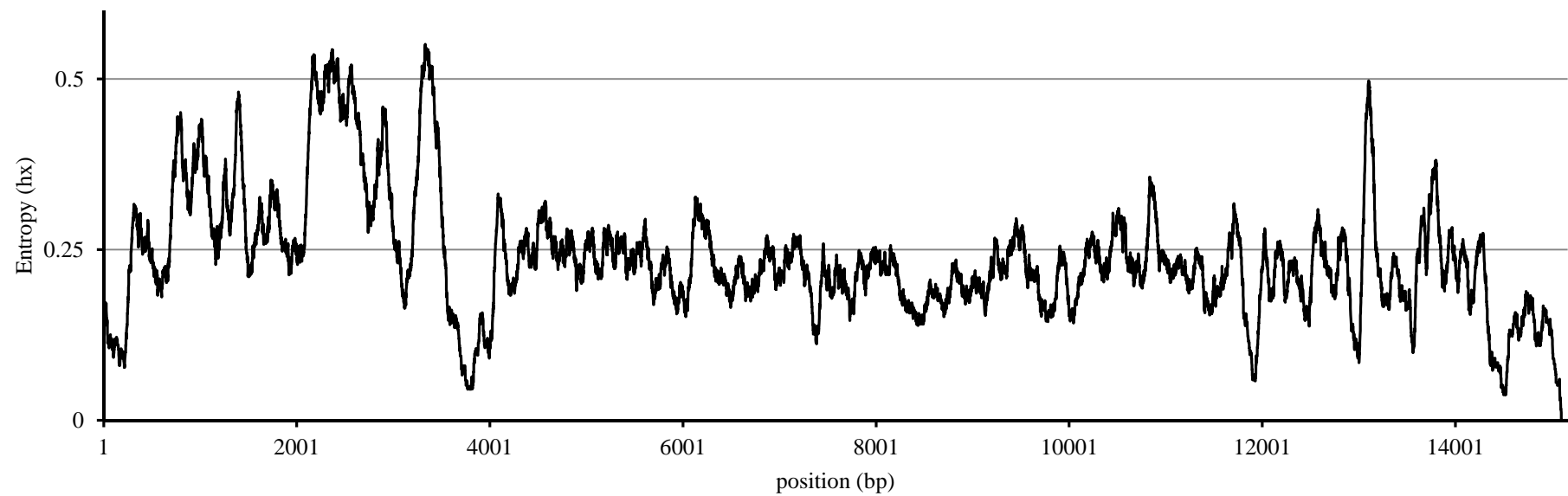

**Figure S2**

Supplement: Additional file 3: Figure S2 — Sequence Entropy of type I European PRRSV Strains. Multiple sequence alignment of the genomes [Olot/91-GenBank:KF203132, GenBank:M96262, GenBank:GU737264, GenBank:A26843, GenBank:GQ461593, GenBank:FJ349261, GenBank:DQ489311, GenBank:JF802085, GenBank:GU047344, GenBank:GU047345, GenBank:AY588319, GenBank:AY366525, GenBank:GU067771, GenBank:EU076704, GenBank:DQ864705] were generated with MUSCLE and entropy analysed with Hyphy [25]. Higher entropies denote regions with higher variability. [file 1743-422X-11-42-S3.pdf]
